# Embryonic thermal environments drive plasticity in gene expression

**DOI:** 10.1007/s10695-025-01522-x

**Published:** 2025-06-10

**Authors:** Anthony A. Snead, Corey R. Quackenbush, Shawn Trojahn, Anna L. McDonald, Luana S.F. Lins, Chris Cornelius, Paula E. Adams, Dengke Ma, Yuying Hsu, Eric Haag, Frédéric Silvestre, Akira Kanamori, Ryan L. Earley, Joanna L. Kelley

**Affiliations:** 1 Department of Biological Sciences, University of Alabama, 300 Hackberry Lane, Box 870344, Tuscaloosa, AL 35487 USA; 2 School of Biological Sciences, Washington State University, 100 Dairy Road, Pullman, WA 99164 USA; 3 Cardiovascular Research Institute and Department of Physiology, University of California San Francisco, San Francisco, CA 94158 USA; 4 Department of Life Science, National Taiwan Normal University, Taipei, 116 Taiwan; 5 Department of Biology and Biological Sciences Graduate Program, University of Maryland, College Park, MD 20742 USA; 6 Laboratory of Evolutionary and Adaptive Physiology, Institute of Life, Earth, and the Environment, University of Namur, 61 Rue de Bruxelles, 5000 Namur, Belgium; 7 Division of Biological Science, Graduate School of Science, Nagoya University, Aichi, 464-8602 Japan; 8 Present Address: Department of Biology, New York University, New York, NY 10003 USA; 9 Present Address: Australian National Insect Collection, CSIRO, Canberra, Australia; 10 Present Address: Department of Biological Sciences, Auburn University, Auburn, AL 36849 USA; 11 Present Address: Department of Ecology and Evolutionary Biology, University of California Santa Cruz, Santa Cruz, CA 95060 USA

**Keywords:** Thermal plasticity, Developmental plasticity, Transcriptomics, Thermal regime

## Abstract

When embryos experience different environments than their parents, plasticity can enable the development of alternate phenotypes that confer higher fitness in the new conditions. Temperature-induced plasticity could be especially critical for species that inhabit areas with considerable thermal variation. We studied transcriptional variation in embryos of mangrove rivulus (*Kryptolebias marmoratus*)—a self-fertilizing hermaphroditic, eurythermal fish that resides in notoriously spatiotemporally variable mangrove forests—exposed to different thermal regimes during development. To study transcriptional plasticity, we first improved the genome assembly to chromosome length scaffolds (N50 of 28.17 Megabases). Whole transcriptome sequencing revealed that both temperature and developmental timing modulated embryonic gene expression. We found few differences in gene expression between embryos incubated in cold and warm conditions and assessed before the temperature-sensitive period of development, indicating high resistance to stochastic changes in gene expression early in development. Replicate embryos exposed to cold temperatures and sampled after the temperature-sensitive period showed less variation in gene expression than those sampled before, suggesting canalization of the plastic response. DNA replication/repair, organelle, and gas transport pathways were upregulated while nervous system development, cell signaling, and cell adhesion were downregulated in cold-exposed compared to warm-exposed embryos sampled after the temperature-sensitive period. These plastic shifts in gene expression could have major implications for reorganizing the phenotype (e.g., apoptosis, mitosis) in response to environmental changes occurring within a generation.

**Supplementary Information:**

The online version contains supplementary material available at 10.1007/s10695-025-01522-x.

## Introduction

An individual’s phenotype arises from the interplay between its genotype, the environment, and their interactions, resulting in traits that range from stable to variable. Invariant traits are insensitive to the environment and show little variance among genetically identical individuals, while variable traits differ stochastically between individuals with the same genotype occupying the same environment (Abley et al. 2016). Plastic traits, however, are highly environmentally dependent, with phenotypic variance being significantly lower among individuals occupying (or raised in) the same environment than in different environments. The expression of plastic traits and their sensitivity to environmental inputs also vary, often predictably, among genotypes (genotype-by-environment interactions). Plasticity has been observed across taxa in response to both biotic and abiotic stimuli such as changes in predator abundance, light, precipitation, and temperature (Hansson 2004; Härer et al. 2017; Mallard et al. 2020; Sentis et al. 2017). Plasticity often is adaptive because it enables relatively rapid responses to environmental change. However, plasticity can also be maladaptive, particularly in cases where environments experienced during development and adulthood are incongruent (Cenzer 2017; Langerhans and DeWitt 2002). In this sense, plasticity can have major evolutionary implications by altering the distribution of phenotypes (e.g., shifting trait values closer to or further away from some theoretical “optimum” with highest relative fitness), thereby protecting alleles from or exposing alleles to natural selection (Fox et al. 2019; Huey et al. 2003). Understanding the mechanisms underlying plastic responses to the environment, which can produce considerable phenotypic diversity within a species, is essential for evaluating species-specific evolutionary responses to environmental change.

Given that climate change will continue to alter thermal regimes (i.e., temporal patterns of temperature change on any scale), exploring phenotypic responses to changes in temperature is particularly timely. Multiple animal taxa have temperature-induced plasticity that contributes to observed phenotypic variation (Gleason et al. 2018; Orizaola and Laurila 2008; Sawall et al. 2015). The ability of an individual to mount a plastic response to temperature often depends on the life stage during which the individual is exposed (Fraimout et al. 2018; Noble et al. 2018; While et al. 2018), often called thermocritical or thermolabile periods. Such plastic responses to temperature can be accompanied by pronounced changes in gene expression that presumably dictate subsequent shifts in the development of the phenotype (Riddell et al. 2019; Zhu et al. 2016). The interaction between thermal regime and developmental stage could thus drive plastic changes in gene expression that have important ecological and evolutionary consequences.

Species within intertidal habitats (e.g., rocky intertidal, mangrove forests) often experience significant variations in water temperature both temporally (Mislan et al. 2009; Morris and Taylor 1983; Wolfe et al. 2020) and spatially (Helmuth et al. 2016; Serôdio and Catarino 1999). Spatial variation in temperature accompanies changes in microhabitat within the intertidal, which can be driven by both abiotic (Denny et al. 2006; Helmuth and Hofmann 2001; Miller et al. 2009) and biotic factors (Guo et al. 2017; Taylor 1992). Therefore, species within intertidal habitats are ideal models in which to explore time-sensitive, temperature-dependent phenotypic plasticity. In these environments, temperature fluctuations across both time and space are ecologically relevant (e.g., can dictate community structure) and likely to become more extreme in the coming decades (Garcia et al. 2014; He and Silliman 2019; IPPC 2014). Moreover, tropical species are currently experiencing more cold extremes, and the incidence is likely to accelerate with time (Williams et al. 2015; Leriorato et al. 2021).

The mangrove rivulus fish (*Kryptolebias marmoratus*; henceforth “rivulus”) is a small, cryptic, eurythermal fish inhabiting New World mangrove forests of Central America, the Bahamas, peninsular Florida (USA), and the Florida Keys (USA) (Tatarenkov et al. 2017; Taylor 2012). Rivulus populations consist mostly of hermaphrodites and very few males (typically < 5% but up to 25% in Twin Cayes, Belize; Davis et al. 1990; Turner et al. 1992). They reproduce primarily through self-fertilization (“selfing”), although rare outcrossing events occur (Harrington 1961; Mackiewicz et al. 2006; Tatarenkov et al. 2015; Tatarenkov et al. 2017). After repeated generations of selfing, rivulus can produce isogenic lineages with offspring nearly genetically identical to both the parent and siblings (Harrington 1968), with some heterozygosity persisting (Lins et al. 2018). Rivulus also are quite sensitive to temperature during embryonic development. When eggs are exposed to 18–20 °C at the beginning of the thermolabile period (embryonic developmental stage 31; Mourabit et al. 2011), a significant proportion of individuals develop into males, with the exact proportion being genotype-dependent (Ellison et al. 2015; Harrington 1968; Turner et al. 2006). Temperature and its interaction with genotype also drive changes in growth rate, with genotypes varying in the extent to which growth rates decrease in low temperatures (Lin and Dunson 1999). Rivulus are often found in warm water (> 20°; Turner et al. 2006) but survive in the wild at temperatures between 7 and 38 °C (Taylor et al. 1995), making temperature a biologically relevant stressor that drives plastic responses. Rivulus is thus an appropriate model in which to investigate time-sensitive, temperature-induced plasticity in gene expression because of the following: (i) they exhibit phenotypically plastic responses to temperature; (ii) these plastic responses are initiated during specific developmental periods; and (iii) isogenic lineages can be used to control for genotype and disentangle environmental effects.

 Using previously developed -omics resources (Kelley et al. 2012; 2016), we investigated how embryonic exposure to divergent thermal regimes alters whole transcriptome patterns of gene expression. We also scaffolded rivulus’ genome, providing a chromosome length reference for studies seeking to investigate the molecular underpinnings of temperature-induced plasticity. We aimed to determine which molecular pathways change in rivulus embryos incubated in cold (20 °C) or warm (25 °C) water, as well as before and after the thermolabile period (stage 31) in each temperature. We hypothesized that variation in gene expression would exist between temperature treatments (20 °C, 25 °C) both before and after the thermolabile period. We predicted that the most dramatic temporal changes in gene expression would correspond to cold temperature treatments, given that both sex and growth rates are significantly altered by low temperature exposure during embryonic development (Ellison et al. 2015; Harrington 1968; Lin and Dunson 1999; Turner et al. 2006).

## Materials and methods

### Experimental design

We used one isogenic lineage of *Kryptolebias marmoratus* collected by D. Scott Taylor from Reckley Hill Lake, San Salvador, Bahamas in 1997 and maintained in the Earley lab since 2008. We focused on this specific lineage (hereafter, RHL) because it was used to generate the reference genome (Kelley et al. 2016; Lins et al. 2018), on which this study expands. Adults were housed individually in ventilated 1.2 L Rubbermaid® containers (TakeAlongs® deep square) filled with 600 ml of 25 parts per thousand (ppt) salt water (aged tap water and Instant Ocean® salt) and maintained on a 12-h light:12-h dark photoperiod. Ambient (air) temperature was 26.2 ± 0.002 °C (mean ± SEM for temperature recordings every 20 min for the study duration); water temperature is typically 1–2 °C cooler than the air temperature. Fish were fed daily with 2 ml brine shrimp (*Artemia*) nauplii suspended in 25 ppt water (~ 500 nauplii/ml). Poly-Fil® was placed in the corner of each container at the air–water interface as egg-laying substrate and was checked twice weekly.

Self-fertilized eggs were collected from each container and photographed with a Canon® G9 camera attached to a Zeiss Stemi 2000-C stereomicroscope to determine the initial developmental stage (Mourabit et al. 2011); fertilized eggs can be retained by the mother for various amounts of time before being laid. Eggs collected prior to developmental stage 20 (Mourabit et al. 2011) were placed individually in 7 ml, 13 × 100 mm polystyrene test tubes filled halfway with 25 ppt saltwater. Tubes were labeled with unique identification numbers and randomly assigned to one of two time points—whether the embryo would be removed *before* or *after* the thermolabile period of development (stage 31 [Harrington 1968; Mourabit et al. 2011]), which we refer to as *Pre* or *Post* critical period, respectively. Embryos also were assigned to water temperature treatments (20 °C or 25 °C; Cold and Warm, respectively), with the warm (25 °C) condition effectively serving as the control because this is the water temperature at which the fish are normally held. Embryos assigned to *Pre* were removed at developmental stages 29 or 30, and those assigned to *Post* were removed at developmental stage 32 (see Mourabit et al. 2011). Time point assignments (*Pre* and *Post*) were crossed in a full-factorial design with temperature treatment to generate four comparison groups: Pre-Cold (PrC), Post-Cold (PoC), Pre-Warm (PrW), Post-Warm (PoW). Tubes containing embryos were then placed haphazardly into weighted Bel-Art™ No-Wire grip racks within the center of a water bath set to the assigned temperature-controlled treatment. A formula was developed based on the timing of development described in Mourabit et al. (2011) to provide an estimate of the date each embryo would reach the thermolabile period (stage 31; Supporting Information (SI): Developmental Formula) using the initial development stage and the number of hours developing. The timing of visual inspections of the eggs was also based on temperature treatment (e.g., embryos in cold water develop slower than embryos in warm water). Thus, embryos were exposed to their respective temperature treatment from the time of egg collection (with documented stage of development) to either *Pre* or *Post*, depending on time point assignment.

### Treatment set-up and treatment details

Treatment tanks of temperature-controlled water were kept circulating by ViaAqua® 350 and Marineland® Maxi-Jet 600 submersible pumps. The Cold tank was kept at 20 °C with an Arctica® Titanium Chiller (1/10 HP). The Warm tank had water pumped in using a Marineland® Maxi-Jet 600 submersible pump from a separate bucket chilled with a NOVA TEC IceProbe® Water Chiller through a Hydor™ ETH In-Line Heater to maintain the temperature at 25 °C. Temperature was monitored with Alpha Mach iBCod 22L temperature data loggers that were placed in a central location within each tank (SI Table 1)*.* Water was added to each tank approximately twice per week to always maintain water levels near the top of the tubes, ensuring eggs were always in treatment. Water temperature in the tanks was measured before and after the replacement of evaporated water to ensure that the temperature did not change more than one degree, which could be regulated by the heaters and chillers quickly. In each tank, an Erlenmeyer flask with 25 ppt salt water was kept at the appropriate temperature to ensure that eggs were immediately exposed to that temperature when first loaded and for each weekly water change.

### Final staging and processing

Once an embryo reached the appropriate developmental stage, estimated using the aforementioned formula (SI), it was gently removed from treatment with a transfer pipette, placed on a petri dish, and photographed with the Canon® G9 camera attached to a Zeiss Stemi 2000-C stereomicroscope. Developmental stage was determined by comparing the photograph to Mourabit et al. (2011). Embryos were transferred directly to a 0.5-ml microcentrifuge tube filled with RNAlater. Labeled microcentrifuge tubes were kept at 4 °C for 24 h then stored at –80 °C.

### Genome improvement

#### Library preparation and sequencing

To improve the existing reference genome, a Chicago library was prepared as described by Putnam et al. (2016). Briefly, ~ 500 ng of High Molecular Weight gDNA (mean fragment length = 75 kilobases (kb)) was reconstituted into chromatin in vitro and fixed with formaldehyde. Fixed chromatin was digested with DpnII, the 5’ overhangs filled in with biotinylated nucleotides, and free blunt ends ligated. After ligation, crosslinks were reversed, and DNA purified from protein was treated to remove biotin external to ligated fragments. DNA was then sheared to ~ 350 basepairs (bp) mean fragment size, and sequencing libraries were generated using NEBNext Ultra enzymes and Illumina-compatible adapters. Biotin-containing fragments were isolated using streptavidin beads before PCR enrichment of each library. Libraries were sequenced on an Illumina HiSeq X to produce 242 million 2 × 150 bp paired-end reads, which provided 176.60 × physical coverage of the genome (1–100 kb). Two Dovetail HiC libraries were prepared as described for the Chicago library (Lieberman-Aiden et al. 2009). The number and length of read pairs produced for each library were as follows: 40 million, 2 × 150 bp for library 1; 178 million, 2 × 150 bp for library 2. Together, these Dovetail HiC library reads provided 86.97 × physical coverage of the genome (10–10,000 kb).

#### Scaffolding the assembly with HiRise

The draft de novo assembly (Rhee et al. 2017), short reads, Chicago library reads, and Dovetail HiC library reads were used as input data for HiRise, a software pipeline designed specifically for using proximity ligation data to scaffold genome assemblies (Putnam et al. 2016). An iterative analysis was conducted. First, shotgun and Chicago library sequences were aligned to the draft input assembly using a modified SNAP read mapper (http://snap.cs.berkeley.edu). The distance between Chicago read pairs mapped within draft scaffolds were analyzed by HiRise to produce a likelihood model for genomic distance between read pairs. The model was used to identify and break putative misjoins, score prospective joins, and make joins. After aligning and scaffolding Chicago data, Dovetail HiC library sequences were aligned and scaffolded in the same way. After scaffolding, shotgun sequences were used to close gaps between contigs.

**Fig. 1:**
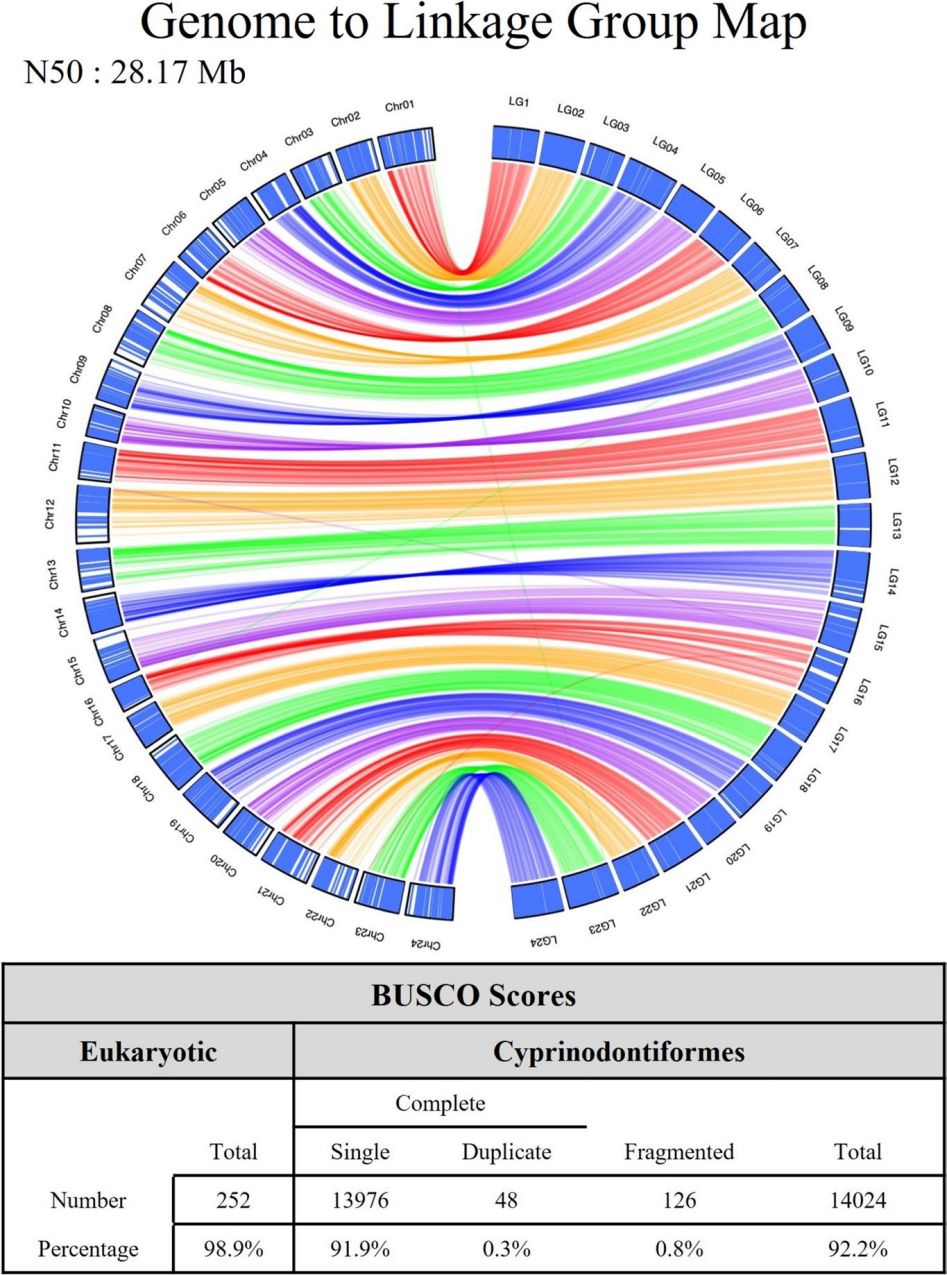
Visualization of the relationship between the improved genome assembly from this study and previously described linkage groups (Kanamori et al. 2016), demonstrating chromosome length scaffolding. The figure includes the N50 (28.17 Mb) and BUSCO-related counts and percentages included for both eukaryotes (98.9%) and Cyprinodontiformes (91.9%)

Completeness of the genome was determined using the program Benchmarking Universal Single-Copy Orthologs (BUSCO) and the general eukaryota_odb10 and specific cyprinodontiformes_odb10 BUSCO databases (Simão et al. 2015). To visualize genome contiguity, genetic markers for the 24 linkage groups identified by Kanamori and colleagues (2016) were mapped to our newly scaffolded genome using BWA (Li and Durbin 2009). The resulting SAM file with a randomly chosen single marker per unique map position was used in R (version 4.0.3) to create the datafile for Fig. 1.

### RNA isolation and RNA-sequencing library preparation

Twelve embryos, three per treatment, were shipped to Washington State University and stored at − 80 °C. RNA was isolated using the Macherey–Nagel NucleoSpin RNA XS kit according to the manufacturer’s instructions. RNA quantity and quality were determined using a Qubit fluorometer and an Advanced Analytical Technologies (AATI) Fragment Analyzer, respectively. High-quality samples were normalized to 500 ng total RNA for input into library preparation. Samples were enriched for mRNA using New England Biolabs NEBNext Poly(A) mRNA Magnetic Isolation Module and prepared for sequencing using NEBNext Ultra Directional RNA Library Prep Kit for Illumina. To determine the molarity of each library, samples were run on an AATI Fragment Analyzer. An equal molar pool was created from all libraries and sequenced on one lane of an Illumina HiSeq 2500 System (paired end, 125 bp reads) at the New York Genome Center. All sequence data were deposited in the Sequence Read Archive on GenBank (BioProject ID: PRJNA401942).

### Read mapping and gene expression matrix

Raw reads generated from the libraries above were adapter trimmed and then quality trimmed using Trim Galore! (v0.4.1) (Krueger 2015). Trimmed reads were subsampled so that each set of paired reads would not exceed a total of 20 million reads. Trimmed reads were mapped to the newly scaffolded *Kryptolebias marmoratus* genome (GenBank Accession: GCF_ 001649575.2) using Hisat2 (version 2.1.0), which was guided by reference annotation (Kim et al. 2015). Resulting SAM files had mate pair information fixed using Picard Tools FixMateInformation (version 2.21.4) and converted to BAM files using SAMtools (version 1.2) view (Li et al. 2009). StringTie (version 1.3.4 d) (Pertea et al. 2015) generated gene counts for each embryo guided by the reference annotations. A Python script included in the StringTie download, prepDE.py, merged gene counts for each embryo into a gene count matrix.

### Gene expression patterns

Henceforth, all treatments are referred to by their acronym: PrC (Pre-Cold), PoC (Post-Cold), PrW (Pre-Warm), PoW (Post-Warm). The gene count matrix was loaded into R (v 4.0.3) (R Core Team 2020) and genes with fewer than 0.5 counts per million in three individuals were filtered out. To visualize similarity or dissimilarity in gene expression patterns between samples, we plotted the top 500, 1000, and 10,000 genes based on log_2_-fold change on a multidimensional scaling (MDS) plot using pairwise comparisons and the package *limma* (Ritchie et al. 2015; SI Figs. 2–4).

Differential expression was quantified using the Bioconductor package *edgeR* (version 3.32.1), implementing a generalized linear model (GLM) (Robinson et al. 2010). To test for statistical significance, we used the GLM quasi-likelihood F-test (glmQLFTest in *edgeR*) with a Benjamini–Hochberg correction. Significance was set at a false discovery rate (FDR) < 0.05. To disentangle how developmental stage, temperature, and their interaction influenced gene expression, we performed a series of pairwise comparisons. To determine gene expression changes that could be related to temperature, we compared PrC to PrW embryos and PoC to PoW embryos. To determine changes in gene expression across development at a specific temperature, we compared PrC to PoC embryos and PrW to PoW embryos.

### Gene ontology and Kyoto encyclopedia of genes and genome enrichment

Gene ontology (GO) analyses were performed for all comparisons with significant differentially expressed genes (PrC vs. PrW, PrC vs. PoC, PoC vs. PoW but not PrW vs. PoW) using the *gprofiler2* package (version 0.2.0) (Kolberg et al. 2020). GO terms were found for three ontologies: biological process, cellular component, and molecular function. To determine which GO terms were significant, a one-sided Fisher’s exact test with FDR < 0.05 was performed separately for significantly upregulated and downregulated genes in each comparison using all annotated genes as the background (e.g., PrC vs. PrW). Similarly, Kyoto encyclopedia of genes and genome (KEGG) enrichment analyses at the level of KEGG ontology and pathway were performed for the same comparisons using *clusterProfiler* (Xu et al. 2024) using a Fisher’s exact test with FDR < 0.05 with a minimum gene set size of five. While *gprofiler2* can also test for KEGG enrichment, the package only uses precompiled data and KEGG was not available for rivulus through the package. *ClusterProfiler* allows for user-defined gene-to-term mapping files. Therefore, *clusterProfiler* was used with all genes found in our expression matrix as the background set with KEGG terms and ontology extracted from the annotation file.

## Results

### Genome scaffolding and annotation

Chicago library preparation and sequencing generated 242 million, 150 bp paired-end reads, providing 263.57 × coverage of the genome. After Chicago sequencing, the reference genome (GCF_001649575.1) was improved from a scaffold N50 of 2.23 Megabases (Mb) to 5.02 Mb. After Dovetail HiRise assembly, the genome was further improved to a scaffold N50 of 28.17 Mb. The total number of scaffolds was reduced from 3073 to 300, including the mitochondrial genome. Of the 299 nuclear scaffolds, 27 contained at least one genetic marker from the previously published genetic map for *K. marmoratus* (Kanamori et al. 2016). Of the 27 scaffolds with at least one marker, more than 99% of the genetic markers were contained within 24 of those scaffolds, indicating that the new genome is scaffolded to chromosome length (Fig. 1), with the additional three scaffolds possibly being difficult-to-assemble regions of the chromosomes. Rivulus has a haploid karyotype of *n* = 24 (Scheel 1972). The newly scaffolded genome had 252 of 255 eukaryotic complete BUSCOs (98.9%). A total of 15,213 Cyprinodontiformes BUSCOs were searched, and there were 14,024 (92.2%) complete BUSCOs, including 13,976 (91.9%) single copy and 48 (0.3%) complete and duplicated, as well as 126 (0.8%) fragmented BUSCOs in the newly scaffolded genome (Fig. 1).

### Embryo RNA

Total trimmed reads ranged from 7,176,142 to 60,088,608 and 7,176,142 to 40,000,000 after subsampling. Mapping percentages ranged from 42.5 to 91.6% (SI Table 2). Two samples from the PrW treatment had low mapping percentages (SI Table 2) that could potentially impact our results. However, the last PrW sample had the highest percent of reads mapped (91.6%; SI Table 2), and our filtering regime removes genes expressed at low levels (less than 0.5 counts per million in three samples). Total counts for differential expression analysis, prior to filtering, ranged from 2,452,255 (RPRW_112) to 35,119,175 (RPRW_39). After filtering for genes expressed at a low level (less than 0.5 counts per million in at least three individuals), 21,447 of the 25,186 genes remained.

### Gene expression patterns

MDS plots for the 500, 1000, and 10,000 genes with the greatest log fold change showed considerable separation between treatments (SI Figs. 1–3), the most prominent being between PoC embryos and embryos from the remaining treatments (PrC, PrW, PoW). Generally, samples from each treatment were distinct from other treatments, regardless of the number of genes selected (500, 1000, 10,000), indicating similar gene expression profiles among replicates. PoC and PrW samples, however, were most highly clustered, suggesting relatively low within-treatment variation compared to PrC and PoW (SI Figs. 1–3).

The vast majority of significantly differentially expressed genes occurred between embryos sampled before and after the thermolabile period in cold temperatures (PrC vs. PoC; 1,313 with ~ 75% being downregulated in PrC relative to PoC) and between embryos sampled after the thermolabile period in the two different temperatures (PoC vs. PoW; 1,570 with an approximately equivalent number of up- and downregulated genes) (Table 1). There were zero significantly differentially expressed genes between embryos sampled before and after the thermolabile period in warm temperatures (PrW vs. PoW), and only 43 between embryos sampled before the thermolabile period in the two different temperatures (PrC vs. PrW) (Table 1, SI Table 3, SI Fig. 4).

**Table 1:** Differential gene expression, Gene ontology (GO), and Kyoto encyclopedia of genes and genomes (KEGG) results for each temperature × developmental stage comparison. All results presented were significant with the false discovery rate corrected p-values < 0.05. An asterisk (*) indicates that the comparison did not include the minimum gene set (5 genes). Direction indicates whether the genes or gene ontology were upregulated (↑) or downregulated (↓); for instance, in the pre-cold vs. pre-warm comparison, the ↑ associated with 10 differentially expressed genes indicates that 10 genes were upregulated in pre-cold embryos compared to pre-warm embryos

Comparison	Direction of gene expression change	Number of differentially expressed genes	GO enrichment results				KEGG enrichment results	
			Biological processes	Cellular component	Molecular function	Total terms	Ontology	Pathway
**Pre-warm vs. post-warm**	↑	0						
	↓	0						
**Pre-cold vs. pre-warm**	↑	10	26	1	12	49	0*	7
	↓	33	44	0	6	50	10	21
**Pre-cold vs. post-cold**	↑	335	139	7	11	157	28	63
	↓	978	75	39	15	129	98	125
**Post-cold vs. post-warm**	↑	716	34	19	20	73	26	133
	↓	854	20	30	18	68	62	101

### Gene ontology and KEGG enrichment

Comparing embryos raised in the *cold* before and after the thermolabile period (PrC vs. PoC), 286 GO terms, 126 KEGG terms, and 188 KEGG pathways were enriched, with 157, 28, and 63 upregulated along with 129, 98, and 125 downregulated in PrC relative to PoC, respectively (Table 1 (see additional details in SI Table 4, SI Table 5, and SI Table 6)). Upregulated GO terms were associated with neural and sensory development (e.g., differentiation of brain regions [forebrain, thalamus, habenula], retina layer formation, neurogenesis), cell division and differentiation (e.g., G2/M transition of mitotic cycle, microtubule binding, cell fate commitment, transcription factor activity), and macromolecule synthesis, particularly nucleic acids. Upregulated KEGG terms included similar functions such as nervous system development (e.g., HES5, POU-domain transcription factors) and cell division (e.g., STAG1/2). Upregulated KEGG pathways included pathways associated with the cell cycle (e.g., p53 signaling pathway, apoptosis). Downregulated GO terms were diverse and related to intracellular signaling (e.g., calcium ion binding, voltage-gated sodium channels, ion transport), cell–cell connectivity (e.g., desmosomes, adhesion, cell junction organization), vision (e.g., photoreceptor activity, cone photoresponse recovery), energy production (e.g., fatty acid biosynthesis and metabolism, glycolytic process), muscle function (e.g., sarcomere, myofibril, Z-disc), and protein and DNA catabolism (e.g., endopeptidase activity, purine metabolism). Downregulated KEGG terms and pathways aligned well with the observed enriched GO terms, with KEGG terms enriched for visual processes (e.g., GRK1) and muscle functions (e.g., PVALB) alongside KEGG pathways such as phototransduction, cell adhesion, and cardiac muscle contraction.

Comparing embryos raised in cold and warm temperatures *before* the thermolabile period (PrC vs. PrW), 89 GO terms, 10 KEGG terms, and 31 KEGG pathways were enriched, with 39, 0, and 7 upregulated along with 55, 10, and 21 downregulated GO terms, KEGG terms, and KEGG pathways, respectively, in PrC relative to PrW. Upregulated GO terms fell into two main categories: epigenetic processes (e.g., demethylase activity, H3K27me3 modified histone binding) and gas transport (e.g., heme biosynthesis, oxygen binding, oxygen carrier activity). Upregulated KEGG pathways showed similar trends with the polycomb repressive complex pathway (related to histone modification) and porphyrin metabolism pathway (related to respiration) significantly upregulated. Downregulated GO terms aligned primarily with sensory processes, specifically in the eye (e.g., photoreceptor activity, phototransduction, sensory perception of light), G-protein-related signal transduction (e.g., β/γ-subunit complex binding, adenylate cyclase signaling pathway, small GTPases [Ras/Rho]), and energy utilization (e.g., phosphagen biosynthesis and metabolism, lactate dehydrogenase activity, response to sugars). Downregulated KEGG terms were related to signal transduction (e.g., UNC-119) and photoreceptors (e.g., gamma-crystallin), while downregulated KEGG pathways were related to eye function (e.g., phototransduction), metabolism (e.g., propanoate metabolism, pyruvate metabolism), and intracellular signaling (e.g., ErbB signaling pathway).

Comparing embryos raised in cold and warm temperatures *after* the thermolabile period (PoC vs. PoW), 141 GO terms, 88 KEGG terms, and 234 KEGG pathways were enriched, with 73, 26, and 133 upregulated, respectively, along with 68, 62, and 101 downregulated in PoC relative to PoW. Upregulated GO terms were predominantly (~ half) associated with DNA replication and repair, and the cell cycle (e.g., telomere maintenance, Ku70:Ku80 complex [DNA repair], recombination, duplex unwinding, cell cycle checkpoint signaling, organelle production). Gas transport (e.g., oxygen binding, hemoglobin complex) also was upregulated in PoC relative to PoW. Upregulated KEGG terms aligned well with the GO term results with terms related to gas transport (e.g., hemoglobin subunit alpha) as well as terms related to the cell cycle (e.g., tubulin alpha, Cdc42). Upregulated KEGG pathways were related to DNA replication and repair (e.g., DNA replication, mismatch repair, nucleotide excision repair) as well as the cell cycle (e.g., p53 signaling pathway, cell cycle). Downregulated GO terms were associated with intracellular and cell–cell signaling (e.g., cation channel activity, calmodulin-dependent protein kinase activity, vesicular or granular secretion, regulation of neurotransmitter levels) and neural function (e.g., neurexin protein binding, pre- and post-synaptic membranes, circadian and locomotor rhythms). Downregulated KEGG terms were related to neural function (e.g., SV2, KChIPs, neurabin, AMIGO proteins) and cell signaling (e.g., PLC-β). Downregulated KEGG pathways included signal pathways (e.g., MAPK signaling pathway, ErbB signaling pathway, ABC transporters).

GO terms, KEGG terms, and KEGG pathways were not evaluated for embryos raised in the *warm* and sampled before and after the thermolabile period (PrW vs. PoW) because there were no differentially expressed genes between the two treatments.

## Discussion

The RNA-sequencing results supported our hypothesis that patterns of gene expression would vary as a function of both temperature (cold vs. warm) and developmental timing (before vs. after thermolabile period). Cold exposure drove significant molecular, cellular, and neural reorganization across development that was lacking in warm-exposed embryos. This temperature-dependent divergence in gene expression was evident after the thermolabile period as well. Cold-exposed embryos invested more in building cells and tissues and transporting oxygen to them, and less in intra- and intercellular communication and central nervous system function than warm-exposed embryos during the final stages of development. We showed a significant number of up- and downregulated transcripts upon cold exposure, which corresponds to findings in another tropical fish (zebrafish; Scott and Johnston 2012) but deviates from the predominant upregulation of genes that occurs with cold exposure in fishes that occupy cooler water (Chen et al. 2008; Gracey et al. 2004). Importantly, variation in gene expression patterns emerged despite using animals from a single isogenic lineage (RHL), which effectively eliminates genotype or genotype-by-environment as sources of molecular variance. The ability to isolate these molecular responses to the environment makes rivulus a useful model to investigate the consequences of environmental change. Future studies, however, should capitalize on the diversity of genotypes that exist within and among populations to determine whether and to what extent our findings are generalizable among lineages. This is particularly important given that rivulus lineages can vary, sometimes considerably, in their critical thermal maxima and in the threshold temperature that elicits emersion from the water (e.g., Currie & Tattersall 2018; Brown et al. 2024). Such variation implies that the scope of plasticity might also differ among lineages; e.g., lower CT_max_ would narrow the scope, and perhaps have implications for how embryo development changes at different temperatures.

Our results reveal considerable plasticity in gene expression within a lineage, especially during cold exposure, and likely driven by a host of epigenetic processes upregulated before the thermolabile period (in PrC relative to PrW) (see also Ellison et al. 2015). This corresponds to our prediction that cold temperatures would drive drastic changes in gene expression, perhaps to reorganize development in ways that produce dramatically different sexual phenotypes, growth rates, and body size relative to those that develop in warmer conditions (Ellison et al. 2015; Harrington 1968; Lin and Dunson 1999; Turner et al. 2006). By also assembling a chromosome-level genome (Fig. 1), we facilitate future research in this area while opening new potential avenues of research and further developing rivulus as a potential model system.

### Temperature regime dependent gene expression patterns

Using a full factorial design, we disentangled the impact of exposure timing and temperature on gene expression patterns across the transcriptome. Because zero genes were differentially expressed between PrW and PoW, warm temperatures are likely a benign environmental scenario. However, differentially expressed genes were observed in all other comparisons (PrC vs. PrW, PoC vs. PoW, and PrC vs. PoC; Table 1). Tight MDS sample clustering of PrW embryos (SI Figs. 2–4) indicates low variability between samples, suggesting that gene expression is tightly regulated in benign conditions before the thermolabile period. Furthermore, PrC vs. PrW treatments had substantially fewer differentially expressed genes (43 genes) relative to the PoC vs. PoW (1570) or PrC vs. PoC (1313) comparisons (Table 1). The transcriptome might thus be more resistant to stochastic or environmentally induced variation in gene expression before the thermolabile period, as previously demonstrated in *Drosophila* during early development (Liu et al. 2020)*.* PrC samples also were tightly clustered in the MDS plots (SI Figs. 2–4), indicating a predictable developmental response to cold even before the thermolabile period.

Thermally induced plasticity in gene expression during development is well documented (Oliver et al. 2013; Metzger and Schulte 2018); however, our data also revealed the importance of exposure timing. The PrC vs. PoC comparison captured changes across the thermolabile period when embryos were incubated in the cold. Cold temperatures altered gene expression regardless of when exposure took place, but exposure during the thermolabile period drove a pulse of change in the transcriptome (Table 1). Epigenetic processes stood out as being strongly modulated by temperature (e.g., in PrC vs. PrW), with cold exposure upregulating genes encoding proteins that both facilitate (e.g., demethylase) and inhibit (e.g., H3K27me3) downstream gene expression. This could deconstruct and reconstruct the epigenetic landscape well after global methylation has plateaued during embryogenesis, which is already quite late in rivulus relative to other fishes (Fellous et al. 2018; Wang & Bhandari 2020). Thus, cold exposure might open up a window, via epigenetic reprogramming, for wholesale changes in the phenotype (e.g., Fellous et al. 2022); for instance, cold-induced development of males in rivulus (Ellison et al. 2015; Harrington 1968).

### Gene ontology and KEGG enrichment

Many of the terms downregulated in PrC relative to PoC also were downregulated in PoC relative to PoW, suggesting that cold temperatures might have the overall effect of slowing development, particularly with respect to cell–cell communication and connectivity, sensory systems, and molecular processes involved in protein and DNA catabolism. While we did not closely monitor the timing of development (and so, cannot capture nonlinear patterns), we knew the stage at which the embryo was collected and the stage when it was removed from the experiment, plus the total hours spent incubating. Embryos incubated in cold took about twice as long to develop as those incubated in warm temperatures (average number of hours per developmental stage; cold: 67.09 ± 13.05, warm: 33.19 ± 5.14). Thus, it is possible that some differentially expressed genes were related to differences between temperature treatments in the absolute time embryos spent incubating.

Interestingly, though, cold-exposed (but not warm-exposed) embryos showed variation across the thermolabile period in expression of genes related to brain and sensory development (higher in PrC than PoC). At the thermolabile period (embryonic developmental stage 31), most organ formation in rivulus is complete (Mourabit et al. 2011). Having more differentially expressed genes before stage 31 (PrC) is thus not entirely unexpected, as those transcripts might ultimately support the final stages of nervous system development. However, warm-exposed embryos showed no such temporal change in gene expression, indicating an interaction between temperature and timing. It is possible that we missed an important transition in transcriptomic activity by sampling warm-exposed embryos too late (e.g., before the thermolabile period but after brain development ceased). Functionally, it could be that cold exposure before the thermolabile period upregulates genes that promote restructuring of the brain and associated signaling processes in ways that have predictable (but perhaps not adaptive) phenotypic outcomes, e.g., behavioral variation. Exposure to low temperatures is associated with decreased short-term memory (Jones et al. 2005) and decreased neuronal growth (Peng et al. 2007) in other species. Rivulus depend on intermittently dry complex microhabitat (Taylor 2012) and often fight with conspecifics (Hsu et al. 2008). Therefore, decreased cognitive and locomotory abilities associated with lower developmental temperatures might disrupt rivulus’ capacity to navigate both its physical and social environments.

## Conclusion

Understanding thermally induced plasticity in gene expression is critical for predicting organismal responses to increasingly unpredictable temperature fluctuations (IPCC 2014). Using an isogenic lineage of rivulus, we controlled for genotype and demonstrated that both temperature and developmental stage at exposure drive plastic changes in gene expression. The transcriptome appeared largely resistant to environmental influences before the thermolabile period, and cold temperatures canalized gene expression compared to warmer conditions. Cold-exposed embryos ultimately (after thermolabile period) showed lower gene expression related to neural function and signaling than warm-exposed counterparts, which could have major implications for the behavioral phenotype and individual fitness. Future research should incorporate time and duration of exposure with longitudinal physiology, morphology, and behavioral studies to elucidate how thermal regimes shape individual phenotypes.

## Supplementary Information

Below is the link to the electronic supplementary material.

Supplementary file1 (HTML 14434 KB)

## Data Availability

Raw RNA Sequence data has been deposited under NCBI BioProject PRJNA401942. This Whole Genome Shotgun project has been deposited at DDBJ/ENA/GenBank under the accession LWHD00000000. The version described in this paper is version LWHD02000000. All code is available at the GitHub repository anthonysnead/Plastic-Gene-Rivulus (https://github.com/anthonysnead/Plastic-Gene-Rivulus).
