# Supplementary material for: Embryonic thermal environments drive plasticity in gene expression
Source: Fish Physiol Biochem. 2025 Jun 10;51(3):111. doi: 10.1007/s10695-025-01522-x (PMC12152062; doi:10.1007/s10695-025-01522-x)
Supplement: Supplementary file 1 — Supplementary file1 (HTML 14434 KB) [file 10695_2025_1522_MOESM1_ESM.html]

Plasticity\_gene\_expression\_SI.knit


Plasticity in Gene Expression in Response to Embryonic Environment
Supporting Information
   

Anthony A Snead1,8, Corey R Quackenbush2, Shawn
Trojahn2, Anna L McDonald2, Luana
Lins2,9 Chris Cornelius1, Paula E
Adams1,10, Dengke Ma3 Yuying Hsu4, Eric
Haag5, Frédéric Silvestre6, Akira
Kanamori7, Ryan L Earley1\*, Joanna L
Kelley2,11\*


Affiliations:    
 
1Department of Biological Sciences, University of Alabama,
300 Hackberry Lane, Box 870344, Tuscaloosa, AL 35487   
2School of Biological Sciences, Washington State University,
100 Dairy Road, Pullman, WA 99164   
3Cardiovascular
Research Institute and Department of Physiology, University of
California San Francisco, San Francisco, California, USA
  
4Department of Life Sciences, National Taiwan Normal
University, Taipei 116, Taiwan   
5Department of Biology and
Biological Sciences Graduate Program, University of Maryland, College
Park, MD 20742   
6Laboratory of Evolutionary and Adaptive
Physiology, Institute of Life, Earth, and the Environment, University of
Namur, 61 Rue de Bruxelles, 5000, Namur, Belgium
  
7Division of Biological Science, Graduate School of
Science, Nagoya University, Aichi 464-8602, Japan
  
8Current address: Department of Biology, New York
University, New York, NY 10003   
9Current address:
Australian National Insect Collection, CSIRO, Canberra, Australia
  
10Current address: Department of Biological Sciences,
Auburn University, Auburn, AL, USA   
11Current address:
Department of Ecology and Evolutionary Biology, University of California
Santa Cruz, Santa Cruz, CA, USA   
   
 Authors
for correspondence:   
 \*Joanna
L. Kelley, Department of Ecology and Evolutionary Biology, University of
California Santa Cruz, Santa Cruz, CA, USA, jokelley@ucsc.edu   
\*Ryan Earley, Department of Biological Sciences, University
of Alabama, 300 Hackberry Lane, Box 870344, Tuscaloosa, AL 35487

Developmental
Formula   
   

The formula to estimate when embryos should be removed from treatment
was derived from a non-linear regression with “developmental stage (DS)”
(1-35, from Harrington 1968) as the predictor and “hours of development”
(0-310, from Mourabit et al. 2011) as the response. The resulting
formula was:

  


Hours of Development ~ -33.04 + 4.37\*DS -
0.07\*DS2 - 0.007\*DS3 +
0.0017\*DS4 + 0.00009\*DS5
   

Upon collecting the egg from its mother, we scored developmental stage
(DS) and, using the above formula, calculated the estimated number of
hours that the embryo had already spent developing and thus, how long it
should remain in treatment before being sampled. We subtracted that
value from 120 (with 120 hours being the time it takes the animal to
develop to stage 29), 140 hours (stage 30), 180 hours (stage 31), 211
hours (stage 32), 240 hours (stage 33), or 310 hours (stage 34)
(Mourabit et al. 2011). This value then was converted to days, and
dictated when we sampled the animals from treatment. We confirmed DS
under a stereomicroscope when the embryo was removed from treatment, and
it was this confirmed DS that was used to determine whether the embryo
was pre- or post-thermolabile period.

  
 
References    

Harrington, Jr RW (1968) Delimitation of the thermolabile phenocritical
period of sex determination and differentiation in the ontogeny of the
normally hermaphroditic fish Rivulus marmoratus Poey. *Physiological
Zoology* 41: 447-460.

Mourabit S, Edenbrow M, Croft DP, Kudoh T (2011). Embryonic development
of the self-fertilizing mangrove killifish *Kryptolebias
marmoratus*. *Developmental Dynamics* 240: 1694-1704.

Supporting
Figures   
   

  
SI
Figure 1 The graph visualizing the non-linear regression with
developmental stage, shortened to “DS” for the equation, as the
predictor (x-axis) and the hours of development as the response
(y-axis). The points are data used to derive the formula, and the line
is the non-linear regression. The equation is displayed in the upper
right corner of the graph for reference.

SI
Figure 2 The multidimensional scaling plot for the 500 genes with the
largest fold change between samples using the first two principal
components. Each group (Pre-Cold, Pre-Warm, Post-Cold, and Post-Warm) is
differentiated by shape and color in the legend.

SI
Figure 3 The multidimensional scaling plot for the 1000 genes with the
largest fold change between samples using the first two principal
components. Each group (Pre-Cold, Pre-Warm, Post-Cold, and Post-Warm) is
differentiated by shape and color in the legend.   
   
   

  
SI
Figure 4 The multidimensional scaling plot for the 10000 genes with the
largest fold change between samples using the first two principal
components. Each group (Pre-Cold, Pre-Warm, Post-Cold, and Post-Warm) is
differentiated by shape and color in the legend.

SI
Figure 5 The four-panel interactive plot is faceted by the comparison
(Post-Cold vs. Post-Warm, Pre-Cold vs. Post-Cold, Pre-Cold vs. Pre-Warm,
Pre-Warm vs. Post-Warm). Each point is a gene, and each panel has the
negative log10 of the false discover rate adjust p-value (log odds
probability) on the y- axis and the log fold change on the y axis. The
dashed grey line is the significance cut off (FDR < 0.05); therefore,
any genes above the line are significantly differentially expressed
between the treatments.

Supporting
Tables

SI
Table 1 The interactive table includes all the temperature measurements
for each treatment ( Warm = 25°C, Cold = 20°C) for all dates. The date
column contains the date (Year-Month-Day) and time
(Hour:Minutes:Seconds). Drastic changes in temperature correspond with
the retrieval of the temperature probes to download data.

SI
Table 2 The table contains read information per sample. Each sample is
identified their sample name along with the number of reads after
trimming, subsampling, and the percentage of the subsampled reads that
were mapped to a gene.

| Sample Name | Trimmed | Subsampled | Percent Mapped (%) |
| --- | --- | --- | --- |
| RPRW\_112 | 8,326,750 | 8,326,750 | 42.50 |
| RPOC\_250 | 8,158,694 | 8,158,694 | 85.97 |
| RPOC\_255 | 7,454,576 | 7,454,576 | 83.69 |
| RPOC\_259 | 7,176,142 | 7,176,142 | 84.10 |
| RPRW\_272 | 14,121,180 | 14,121,180 | 45.82 |
| RPOW\_283 | 8,788,552 | 8,788,552 | 84.39 |
| RPOW\_285 | 10,008,752 | 10,008,752 | 90.14 |
| RPRC\_28 | 60,088,608 | 40,000,000 | 90.83 |
| RPRC\_38 | 50,075,722 | 40,000,000 | 90.09 |
| RPRW\_39 | 49,014,042 | 40,000,000 | 91.60 |
| RPOW\_58 | 9,886,540 | 9,886,540 | 83.99 |
| RPRC\_9 | 11,685,830 | 11,685,830 | 83.95 |

SI
Table 3. The interactive table contains all the differential gene
expression results. Each row contains the gene being analyzed, the gene
ID, the log fold change, the log Counts per Million (CPM), the false
discovery corrected p value (FDR), the comparison (Post-Cold
vs. Post-Warm, Pre-Cold vs. Post-Cold, Pre-Cold vs. Pre-Warm, Pre-Warm
vs. Post-Warm), the associated gene ontologies, and descriptions. The
table is grouped by the gene because a gene can have multiple
descriptions and gene ontologies. The groups are collapsible by clicking
on the group row heading. All columns are searchable.

SI
Table 4 The interactive table includes the fisher’s exact test gene
ontology enrichment results with the comparison, false discovery rate
corrected p. value (FDR), term ID, Source, and term name. Term ID refers
to the numerical code for the term name, while term name is the gene
ontology. Source is the gene ontology the term belongs too (Biological
Process, Molecular Function, Cellular Component).

SI
Table 5 The interactive table includes the fisher’s exact test Kyoto
Encylopedia of Genes and Genomes ontology enrichment results with the
comparison, false discovery rate corrected p. value (FDR), term ID, gene
ratio, background ratio, rich factor, fold enrichment, and description.
Term ID refers to the numerical code for the term name.

SI
Table 6 The interactive table includes the fisher’s exact test Kyoto
Encylopedia of Genes and Genomes pathway enrichment results with the
comparison, direction, category, subcategory, false discovery rate
corrected p. value (FDR), term ID, gene ratio, background ratio, rich
factor, fold enrichment, and description. Term ID refers to the
numerical code for the term name.
